# Asymptomatic Coccidioidal Meningitis Relapse: A Demon in Disguise

**DOI:** 10.1177/23247096231175439

**Published:** 2023-05-16

**Authors:** Lovedip Kooner, Arash Heidari, Royce Johnson

**Affiliations:** 1 Kern Medical, University of California, Bakersfield, USA; 2 Valley Fever Institute, Bakersfield, CA, USA

**Keywords:** coccidioidal meningitis, coccidioidomycosis, meningitis, valley fever, coccidioidal meningitis relapse, meningitis relapse

## Abstract

Coccidioides spp is a soil-dwelling, dimorphic fungus that causes coccidioidomycosis. It is endemic to the western hemisphere. Although primarily a respiratory disease, it can also cause a myriad of clinical manifestations, from asymptomatic disease to meningitis. In fact, Coccidioides species is probably the most common etiologic agent of long-term meningitis in California and Arizona. Early diagnosis and treatment are critical to avoid fatal complications. With treatment, the cerebral spinal fluid analysis may return to normal. Relapse of coccidioidal meningitis is usually suspected with recurrence of meningitis symptoms. The patient is a 53-year-old man with a 2-decade history of coccidioidal meningitis who was diagnosed with an asymptomatic relapse of coccidioidal meningitis.

## Introduction

Meningitis is recognized as the most serious type of disseminated coccidioidomycosis and if not treated is lethal.^1^ This form of the disease requires prompt diagnosis and proper management by experts, which reduces the risk of serious complications such as hydrocephalus, vasculitic infarctions, cranial neuropathy, arachnoiditis, and death.^2^

The Infectious Diseases Society of America’s coccidioidal guidelines indicate analyzing cerebral spinal fluid (CSF) for initial diagnosis if meningeal symptoms are present and recommend initial therapy with fluconazole 400 to 1200 mg.^3^ The current medical treatment is thought to only suppress the disease.^4^ Thus, treatment is for life, and if initial treatment fails, another oral triazole or intrathecal Amphotericin B therapy is indicated.^3^ Relapse is common if treatment is stopped.^5^ Clinical, CSF, and radiographic parameters should be used regularly to monitor treatment.^3^ This patient is a 53-year-old man with a 2-decade history of coccidioidal meningitis who was diagnosed with asymptomatic relapsed coccidioidal meningitis.

## Methods

This is a retrospective case review that is approved by Kern Medical Institutional Review Board. Literature search was performed from PubMed and Google Scholar for coccidioidal meningitis relapse, therapeutic drug monitoring, and coccidioidomycosis.

## Objectives

- Coccidioidal meningitis is a complicated disease and can manifest as an asymptomatic relapse.
- Periodic lumbar punctures may indicate a relapse prior to symptom onset.
- Therapeutic drug monitoring may be helpful in determining adherence versus treatment failure as a problem.

## Case Presentation

A 43-year-old man had been diagnosed with coccoidal meningitis for two decades. His course was complicated by hydrocephalus and therefore underwent placement of a ventriculoperitoneal (VP) shunt. His treatment was initiated on fluconazole 1000 mg daily. His care was complicated by multiple VP shunt revisions: the last episode was 7 years prior.

**Figure 1.**
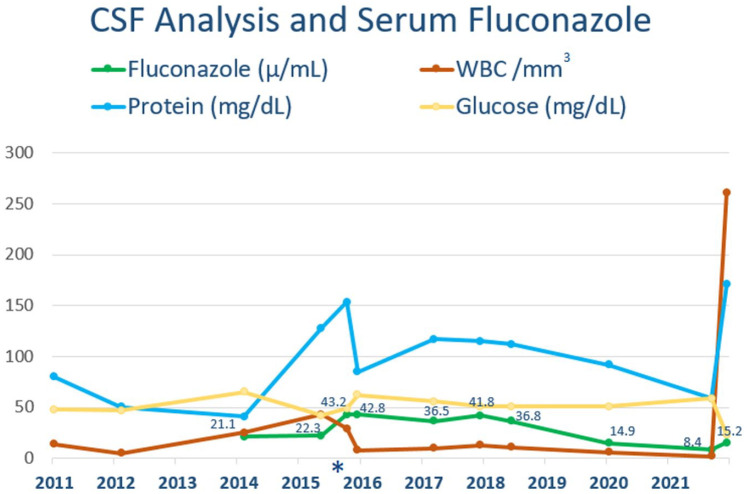
Comparison of LCSF levels of WBC, glucose, and protein with noncontemporaneous serum fluconazole, from 2011 to 2022. The subset of values within the graph indicate the fluconazole levels. *VP Shunt Revision.

Fluconazole levels were monitored at therapeutic goal levels of 40 to 80 µg/mL. Lumbar cerebral spinal fluids (LCSF) were obtained periodically (Figure 1) to monitor his response and showed minimal pleocytosis between 8 to 10, normal protein and glucose, and coccidioidomycosis complement fixation (CF) titers of <1:1 repeatedly.

Periodically during the course of his care, he became nonadherent with medications and visits. He presented for a routine follow-up after a year and a half. At that visit, he admitted to being off of therapy for about 77 months as he felt “great.” A lumbar puncture was performed even though he was entirely asymptomatic. His LCSF showed white blood cell (WBC) of 261 µg/mL, 80% lymphocytic, glucose of 23 mg/dL, protein of 171 mg/dL, and LCSF coccidioidomycosis CF titer of 1:8, indicating a flagrant asymptomatic relapse. Medication compliance was reinforced. Subsequent fluconazole levels were in the therapeutic range, and lumbar LCSF levels improved.

## Discussion

Treatment with high-dose oral fluconazole may achieve remission of coccidioidal meningitis; however, after discontinuation of therapy, there is a high incidence of relapse.^6^ Thus, coccidioidal meningitis requires life-long treatment as currently understood.

Guidelines indicate that after a diagnosis and initiation of treatment for coccidioidal meningitis, LCSF analysis is recommended if the patient has meningeal symptoms, most notably headache.^3^ Most cases of coccidioidal meningitis relapse are symptomatic. It is uncertain the exact number of asymptomatic relapses that occur; however, the Valley Fever Institute (VFI) has experienced this before. The guidelines do not address potential asymptomatic relapse that may be a precursor to symptomatic relapse. Therefore, the VFI has routinely analyzed LCSF on a periodic basis. This and therapeutic drug monitoring allow differentiating between treatment failure and nonadherence. In cases of treatment failure, this can lead to the escalation of therapy via dose increases or changes in medication. In cases like the one presented, it provides a tool to communicate to an asymptomatic patient that their disease is progressing. Furthermore, it prompts the physician to monitor the disease more closely.

Close follow-up is essential to assure maintaining response to therapy and detection of treatment failures and relapses. Therapeutic drug monitoring may lead to a suspicion of nonadherence or therapeutic failure. Lumbar puncture even in an asymptomatic patient can confirm or refute that concern. This case demonstrates that standardized, periodic lumbar punctures and therapeutic drug monitoring may be useful adjuncts for preventing relapse.

## Conclusion

Active coccidioidal meningitis most commonly produces meningeal symptoms. This case demonstrates that even after 2 decades of coccidioidal meningitis treatment, relapse can still occur and life-long treatment is recommended. Relapse may be symptomatic or asymptomatic. This suggests the need for a standardized approach to monitoring disease even in asymptomatic patients that includes periodic evaluation of CSF and therapeutic drug monitoring to reduce morbidity and mortality of relapsed cocciodioidal meningitis. Therapeutic drug monitoring can also assist in differentiating between treatment failure and nonadherence.
